# Compositional Shifts in the Mycobiota of ‘Shine Muscat’ Grape (*Vitis labruscana* Baily × *V. vinifera* L.) Bunches During Cold Storage at Different Temperatures

**DOI:** 10.3390/foods14071169

**Published:** 2025-03-27

**Authors:** Wei Li, Linjun Xie, Yongmei Zhou, Xian Ji, Haijun Wang, Liting Pang, Feicui Liang, Guo Cheng, Jin Zhang

**Affiliations:** 1 Grape and Wine Research Institute, Guangxi Academy of Agricultural Sciences, Nanning 530007, China; liweijt@gxaas.net (W.L.); xielinjun666@163.com (L.X.); zhouyongmei@gxaas.net (Y.Z.); spicadeyx@163.com (L.P.); ll111388@163.com (F.L.); 2 Guangxi Academy of Agricultural Sciences, Nanning 530007, China; ashimawhj@163.com; 3 Institute of Agricultural Products Preservation and Processing Technology, Tianjin Academy of Agricultural Sciences, Tianjin 300384, China; jisandy001@126.com; 4 Key Laboratory of Storage of Agricultural Products of the Ministry of Agriculture and Rural Affairs, Tianjin 300384, China

**Keywords:** *Vitis*, fungal diversity, high-throughput sequencing, post-harvest

## Abstract

The cultivation of ‘Shine Muscat’ grapes is rapidly expanding in East Asia due to their desirable qualities and muscat flavor. Studies have revealed that storing these grapes at an controlled freezing-point temperature diminishes their muscat flavor, whereas storage at 10 °C preserves it. However, the impact of a higher storage temperature on the evolution of microbial communities remains unclear. This study aimed to analyze the mycobiota dynamics of ‘Shine Muscat’ grape bunches under different cold storage temperatures. A total of 1,892,842 and 1,643,200 sequences were obtained from berries and pedicels, identifying over 208 fungal genera from 6 phyla. *Xylariaceae* was the most abundant family, with a prevalence between 7.21% and 69.27% across all sample groups. The primary genera included *Zygosporium*, *Cladosporium*, *Aspergillus*, *Acremonium*, *Podosordaria*, *Zasmidium*, *Penicillium,* and *Alternaria.* Spoilage-related fungi varied with storage temperature, with *Aspergillus*, *Penicillium*, and *Alternaria* being dominant at 0 °C and *Cladosporium*, *Aspergillus*, *Penicillium*, and *Alternaria* being prevalent at 10 °C. The fungal profiles of berries and pedicels differed significantly, and storage temperature further influenced these variations. Our findings highlight distinct fungal diversity and spoilage patterns in ‘Shine Muscat’ grape bunches from the Nanning region compared to those grown in temperate areas, revealing the unique microbial evolution of grape bunches stored at different temperatures in Nanning.

## 1. Introduction

Table grapes are a popular fresh fruit worldwide. In 2019, China became the largest grape producer, with 726.20 thousand hectares of vineyard land and 1.431 × 10^7^ tons of production (National Bureau of Statistics, People’s Republic of China, 2019 http://www.stats.gov.cn/, accessed on 30 October 2024). The ‘Shine Muscat’ is particularly prized in several Chinese regions, but its concentrated ripening period can lead to market saturation. To address this, strategies like production period regulation and cold storage are used to control the timing of grape market entry [1,2].

Grapes, as a non-climacteric fruit, have low post-harvest physiological activity, with perishability mainly driven by microbial decay, weight loss, softening, color degradation, and stem dehydration and darkening [3,4]. Spoilage microorganisms, including *Mucor* spp., Pers. and *Alternaria alternata*, contribute substantially to post-harvest losses, infecting grape bunches at both pre- and post-harvest stages [5]. The microbial community in grapes is shaped by factors like temperature and geographical location [6]. Most research on grape microbiomes has focused on temperate regions and wine production [7,8,9]. Identifying the primary fungal species on stored grape bunches is vital for improving post-harvest disease management [10,11,12]. Despite advancements in preservation technologies, the microbial compositions on stored grape bunches remain elusive.

The mycobiota refers to the taxonomically and functionally diverse community of fungi inhabiting specific environments, host organisms, or ecosystems. These fungal communities play pivotal roles in plant life by interacting with hosts in ways that range from parasitic to mutualistic [13]. While fungi are often recognized as plant pathogens, many symbiotic species contribute significantly to plant functions, including nutrient uptake, stress tolerance, and biocontrol [14,15,16]. Early studies on plant-associated fungal and bacterial communities relied on culture-based techniques and low-resolution molecular methods [17,18,19]. These approaches were not able to distinguish microbial strains accurately or analyze community structure and succession, potentially overlooking up to 95% of microbial diversity in some samples [20,21]. Recently, DNA metabarcoding has emerged as a robust and accessible tool for exploring microbial diversity. This technique typically involves the amplification of barcode regions, such as relatively short DNA sequences (e.g., the ITS region in fungi), using taxon-specific primers, followed by high-throughput sequencing technology to identify a large number of organisms from environmental samples [22]. These techniques have revealed microbial community dynamics in berries, juices, wines, and other foods [23,24,25]. While some research has examined the impact of cultivation methods on microbial communities in grape bunches [6,23], studies on microbial communities in cold-stored grapes are limited. Most existing research on cold-stored grapes has focused on fruit quality and post-harvest disease prevention [26,27,28].

The Guangxi region of China is renowned for its twice-yearly grape production, with early-ripening harvests in both summer and winter. ‘Shine Muscat’ (*Vitis labruscana* Baily × *V. vinifera* L.) is the region’s most popular grape variety. To capitalize on specific holiday markets and maximize profits, these grapes are often stored immediately after harvest to maintain freshness. Investigating the microbial communities on grape bunches could improve grape storage quality. While research on the mycobiota of ‘Shine Muscat’ grapes has primarily focused on the relationship between soil rhizosphere fungi and their effects on plant health and fruit quality [29,30,31], studies focusing on the fungal communities on grape bunches themselves are scarce. This study used high-throughput sequencing to explore the microbial diversity and dominant microorganisms on post-harvest ‘Shine Muscat’ grape from the Guangxi region during short-term cold storage. It also evaluated how storage temperature affects microbial communities. The findings are expected to provide valuable insights for enhancing the post-harvest quality of ‘Shine Muscat’ grapes.

## 2. Material and Methods

### 2.1. Sample Collection and Treatments

‘Shine Muscat’ grape bunches, grown without pre-harvesting fungicide treatment, were collected from the Zhencheng Agriculture Co., Ltd. vineyard (108°8′33″ E, 23°11′48″ N) in Nanning, Guangxi, China, on 13 July 2020. Healthy bunches, each weighing approximately 500 g and exhibiting uniform maturity, with a total soluble solid content of at least 17 °Brix, were randomly sampled in the field. During the picking process, operators wore disposable sterile latex gloves and used fruit-picking shears sterilized by high-temperature moist-heat sterilization to ensure sanitary conditions. The collected grape bunches were immediately placed into disposable sterile polyethylene (PE) sampling bags (Changde BKMAM Biotechnology Co., Ltd., Changde, China) and transported to the laboratory within 2 h to maintain sample integrity. Upon arrival, grape bunches were sorted for quality and placed in unsealed PE, unperforated, fresh-keeping bags (0.03 mm thickness, 40 cm × 60 cm) from the Plastic Industry Technology Development Center, Jiuwang Technology Co., Ltd., Taichuang, China, with two bunches per bag. Each storage box (45 cm × 60 cm × 20 cm, Nanning Cailiang Carton Co., Ltd., Nanning, China) contained two bags. The bunches were then divided into two groups (Group 0 °C and Group 10 °C, each with 36 bunches) and pre-cooled at their respective temperatures for 24 h. After pre-cooling, the bags were air-evacuated and sealed, and stored in two incubators (LRH-500CA, Shanghai Yiheng Scientific Instrument Co., Ltd., Shanghai, China), maintaining storage temperatures of −1 to 1 °C and 9 to 11 °C, respectively, with a 10 cm spacing between boxes to allow airflow. Samples were collected at 0, 3, 7, 21, 35, and 63 days post-treatment. On each sampling date, approximately 1 kg of intact berries with attached pedicels were removed using sterilized scissors. The berries (Group 0-B and 10-B) and pedicels (Group 0-P and 10-P) were carefully separated, frozen with liquid nitrogen, ground into powder under sterile conditions, and stored in sterile 50 mL centrifuge tubes (Changde BKMAM Biotechnology Co., Ltd., Changde, China) at −80 °C for subsequent analysis. All handling was conducted within a vertical flow clean bench (Suzhou Antai Airtech CO., Ltd., Suzhou, China), with operators wearing disposable sterile gloves (Haimen Yangzi Medical Devices Co., Ltd., Haimen, China) at all times. All instruments were rigorously sterilized prior to use.

### 2.2. Berry Drop Rate and Decay Rate Determination

Berry drop and decay rates were measured using the methodology outlined by Gomes et al. [32]. The rates were calculated as follows:

$$\mathrm{Berry}\;\mathrm{drop}\;\mathrm{rate}=\frac{\mathrm{Dropped}\;\mathrm{berry}\;\mathrm{weight}}{\mathrm{Total}\;\mathrm{weight}}\;\times \;100\%$$

$$\mathrm{Berry}\;\mathrm{decay}\;\mathrm{rate}=\frac{\mathrm{Decayed}\;\mathrm{berry}\;\mathrm{weight}}{\mathrm{Total}\;\mathrm{weight}}\;\times \;100\%$$

### 2.3. DNA Extraction, PCR, and Sequencing

Genomic DNA was extracted from 100 mg of powdered sample using the QIAamp DNA Mini Kit (Qiagen, Hilden, Germany), following the manufacturer’s instructions. The internal transcribed spacer (ITS) regions were amplified using the primer pairs ITS1F (5′-CTT GGT CAT TTA GAG GAA GTAA-3′) and ITS2 (5′-GCT GCG TTC TTC ATC GAT GC-3′). PCR reactions were conducted in triplicate in a 20 μL mixture containing 4 μL of 5 × FastPfu Buffer, 2 μL of 2.5 mM dNTPs, 0.8 μL of each primer (5 μM), 0.4 μL of FastPfu Polymerase (All PCR reagents were purchased from Beijing TransGen Biotech Co., Ltd., Beijing, China), and 10 ng of template DNA on a GeneAmp 9700 thermocycler (ABI, Foster city, CA, USA) with the following program: 3 min of denaturation at 95 °C, 33 cycles of 30 s at 95 °C, 30 s for annealing at 55 °C, and 45 s for elongation at 72 °C, and a final extension at 72 °C for 10 min. The PCR products were then extracted from a 2% agarose gel, purified using the AxyPrep DNA Gel Extraction Kit (Axygen Biosciences, Union City, CA, USA), and quantified using QuantiFluor™-ST (Promega, Madison, WI, USA) according to the manufacturer’s protocol. Library construction was performed using the NEXTFLEX^®^ Rapid DNA-Seq Kit (Bioo Scientific, Austin, TX, USA)on purified PCR products through the following steps: (1) adapter ligation; (2) removal of self-ligated adapter fragments using magnetic beads; (3) PCR amplification of adapter-attached library template; and (4) PCR product recovery using magnetic beads to obtain the final library. Sequencing was conducted on the Illumina MiSeq 250 sequencing platform (Illumina, San Diego, CA, USA) at Shanghai Meiji Biomedical Technology Co., Ltd (Shanghai, China). Raw sequencing reads were deposited into the NCBI Sequence Read Archive (SRA) under accession number PRJNA1188757.

### 2.4. Data Processing

An in-house Perl script was utilized to de-multiplex raw FASTQ files, followed by quality filtering using fastp (version 0.19.6) [33] and read merging with FLASH (version 1.2.7) [34]. Reads were truncated where the average quality score fell below 20 over a 50 bp sliding window, and those shorter than 50 bp or containing ambiguous bases were excluded. Only overlapping sequences longer than 10 bp with a maximum mismatch ratio of 0.2 were assembled, while unassembled reads were discarded. Samples were differentiated using barcodes and primers, with exact barcoding matching and up to two mismatches allowed for primers. The processed sequences were then clustered into operational taxonomic units (OTUs) using UPARSE 11 [35] at a 97% sequence similarity threshold, with the most abundant sequence in each OTU selected as the representative. The OTU table was manually curated to remove chloroplast sequences. To mitigate the impact of sequencing depth on alpha and beta diversity metrics, the number of ITS gene sequences per sample was rarefied to 30,266 to standardize the sequencing depth, achieving an average Good’s coverage of 99.97%. Taxonomic assignment of OTU representative sequences was performed using RDP Classifier (version 2.2) [36] against the Unite database project (http://unite.ut.ee/index.php, accessed on 15 April 2024) with a confidence threshold of 0.7.

### 2.5. Statistical Analysis

Bioinformatic analysis of the ‘Shine Muscat’ grape bunch mycobiota was carried out using the Majorbio Cloud platform (https://cloud.majorbio.com, accessed on 15 April 2024). Based on out data, rarefaction curves and alpha diversity indices, including observed OTUs, Chao1 richness, Shannon index, and phylogenetic diversity index, were calculated with Mothur v1.30.1 [37]. Good’s coverage analysis was used to estimate sequence coverage, with OTUs (97% similarity) analyzed using the furthest neighbor approach. The similarity among the fungal communities in different samples was determined by principal coordinate analysis (PCoA) based on Bray–Curtis dissimilarity using the Vegan v2.5-3 package. The PERMANOVA test was used to assess the percentage of variation explained by the treatment along with its statistical significance, using the Vegan v2.5-3 package. The linear discriminant analysis (LDA) effect size (LEfSe) (http://huttenhower.sph.harvard.edu/LEfSe, accessed on 15 April 2024) was calculated to identify the significantly abundant fungal genera among the different groups (LDA score ≥ 2, *p* < 0.05) [38]. The physicochemical indices were analyzed using SPSS17.0 software (SPSS Inc., Chicago, IL, USA). One-way analysis of variance with Duncan’s multiple range test was applied to compare means at a significance level of 5%. Results were expressed as mean ± SD (*n* = 3).

## 3. Results

### 3.1. Stored Berry Morphology and Physiological Indexes

**Figure 1:**
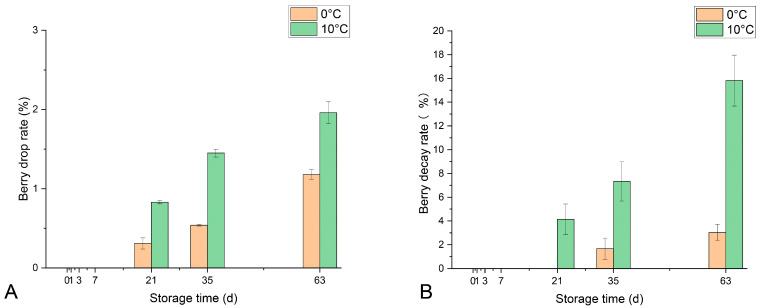
The berry drop rate (**A**) and decay rate (**B**) of ‘Shine Muscat’ grape bunches stored at 0 °C and 10 °C without fungicide treatment over a-nine week period. Vertical bars represent the means’ standard deviation (SD) (*n* = 3).

As shown in Figure 1A, the berry drop rate remained consistently low (below 2%) throughout the 63-day storage period, with Group 0 °C exhibiting a significantly lower drop rate than Group 10 °C. Similarly, Figure 1B shows that the decay rate in Group 0 °C was significantly lower than in Group 10 °C at 21, 35, and 63 days.

### 3.2. Overall Analysis of Sequencing

A total of 1,892,842 high-quality sequences were obtained from berries (Groups 0-B and 10-B) and 1,643,200 from pedicels (Groups 0-P and 10-P). Berry samples in Groups 0-B and 10-B had sequence counts between 43,198 and 66,124 (average: 52,579) with a phylum-level classification rate of 99.94%. Pedicel samples in Groups 0-P and 10-P ranged from 30,371 to 69,617 sequences per sample (average: 45,644), achieving a 99.99% classification rate at the phylum level. These results confirmed that the Illumina sequencing method was well-designed and sufficient for analyzing fungal diversity in these samples (Table S1). Figure S1 presents the rarefaction curves of the 24 groups (with three replicates per group) generated at a cutoff of 0.03. As shown in Figure S1, all curves approached plateaus, suggesting that the sequencing depths were sufficient to capture the entire microbial diversity within each sample.

### 3.3. Fungal α-Diversity

**Figure 2:**
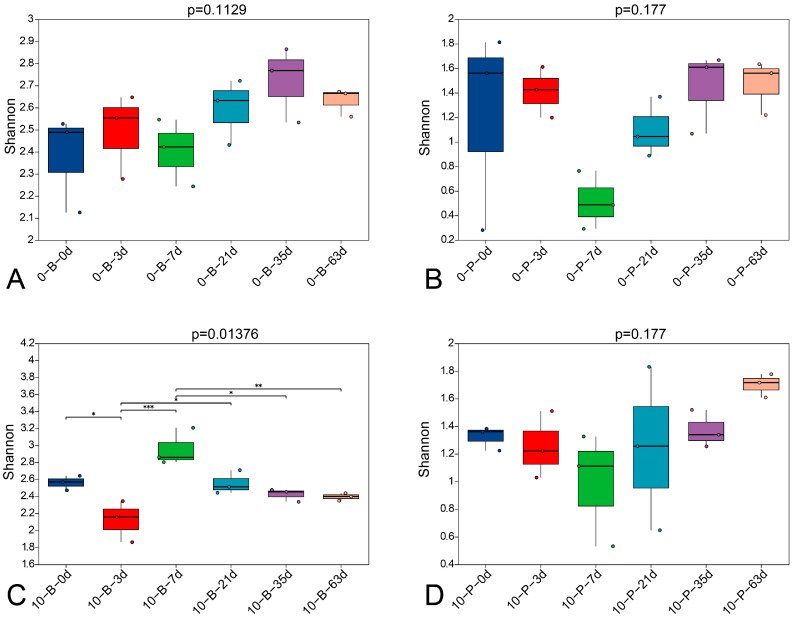
Box plot showing the Shannon index of fungal communities in ‘Shine Muscat’ grape bunches stored at 0 °C and 10 °C without fungicide treatment over nine weeks. Bars represent means ± standard deviation (*n* = 3). The marks *, **, and *** represent significant differences at *p* < 0.05, *p* < 0.01, and *p* < 0.001, respectively. (**A**) berry samples stored at 0 °C; (**B**) pedicel samples stored at 0 °C; (**C**) berry samples stored at 10 °C; (**D**) pedicel samples stored at 10 °C.

The fungal diversity in grape berry and pedicel samples was evaluated using multiple ecological indices (Table S2). All qualified sequences were grouped into OTUs at a 3% distance level to estimate the fungal communities’ phylogenetic diversities. Grape berry samples showed a higher number of OTUs than pedicel samples over the same period. Consistent with the OTU data, diversity indices (Shannon, Ace, PD, and Chao1) also indicated greater fungal diversity in grape berry samples. Good’s coverage value exceeded 0.999 in all samples, indicating that the majority of fungal species were detected. Figure S1 illustrates the rarefaction curves for the 72 samples at a cutoff of 0.03 for fungal communities. As shown in Figure S1, all curves reached a plateau, indicating that the sequencing depth was sufficient to capture the full microbial diversity in each sample. Figure 2 presents the results of the alpha diversity difference test among fungal communities across different storage times, as assessed by the Kruskal–Wallis H test for the Shannon index. No significant variation in the Shannon index was observed during the entire storage period in Groups 0-B, 0-P, and 10-P, whereas a significant variation was noted only in Group 10-B.

### 3.4. Taxonomic Composition

**Figure 3:**
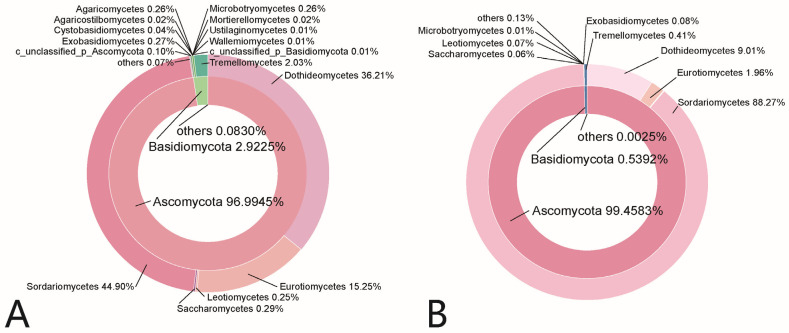
Sunburst chart depicting the total relative abundance of fungal phyla (inner circle) and their corresponding classes (outer circle) across berry samples (**A**) and pedicel samples (**B**).

A total of 379 OTUs across 24 groups (three replicates per group) were classified into 6 phyla, 129 families, and 208 genera (including unclassified). At the phylum level (Table 1), Ascomycota dominated all samples, comprising 96.99% of detected sequences in berries and 99.46% in pedicels (Figure 3). Basidiomycota followed, with 2.92% in berry samples and 0.54% in pedicels. Within Ascomycota, the predominant classes were Dothideomycetes, Eurotiomycetes, and Sordariomycetes. Basidiomycota were mainly represented by Tremellomycetes and Exobasidiomycetes.

**Figure 4:**
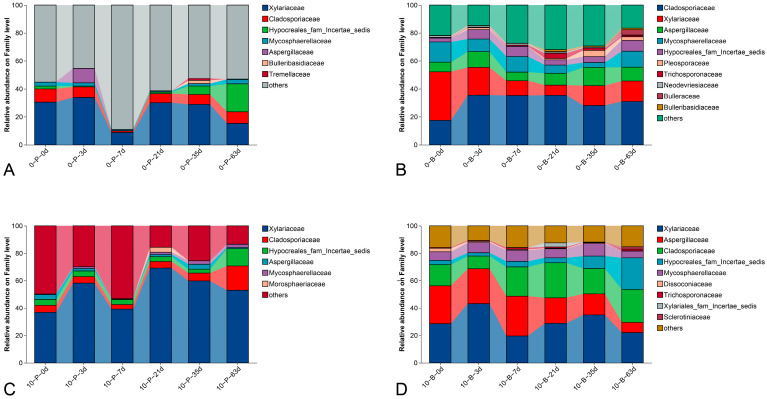
Composition of fungal communities at the family level, showing relative read abundances of different fungal families within each community. Families with relative abundances below 1% are grouped as ‘others’. (**A**) Pedicel samples stored at 0 °C; (**B**) berry samples stored at 0 °C; (**C**) pedicel samples stored at 10 °C; (**D**) berry samples stored at 10 °C.

At the family level, 12 classified families (abundance > 1%) were shared among 129 families in all samples (Figure 4), namely, *Xylariaceae*, *unclassified_c_Sordariomycetes*, *Cladosporiaceae*, *Aspergillaceae*, *Hypocreales_fam_Incertae_sedis*, *Mycosphaerellaceae*, *Trichosporonaceae*, *Pleosporaceae*, *Neodevriesiaceae*, *Trueperaceae*, *Bulleribasidiaceae*, *Dissoconiaceae*, *Bulleraceae*, *Morosphaeriaceae*, *Xylariales_fam_Incertae_sedis*, *Tremellaceae*, *Sclerotiniaceae*, and *unclassified_p_Ascomycota*. *Xylariaceae* was the most abundant family, with relative abundance ranging from 7.21% to 69.27%, while *Gemmatimonadaceae* followed with 1.20% to 35.74% abundances.

**Figure 5:**
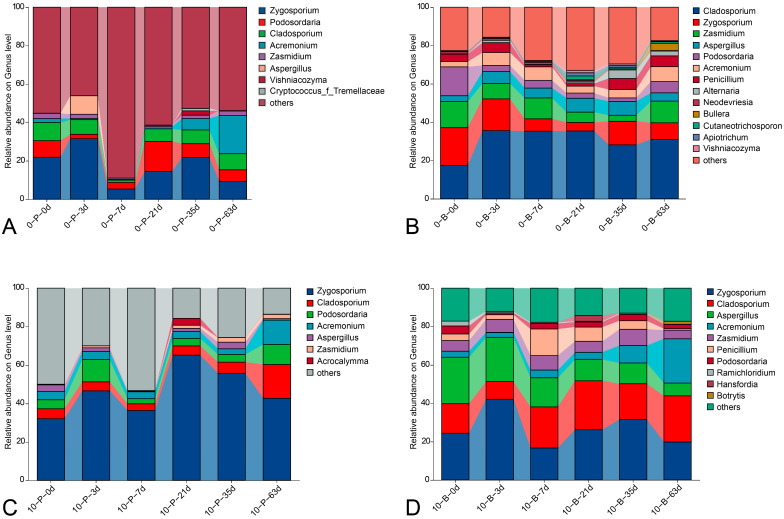
Composition of fungal communities at the genus level, presenting relative read abundances of different fungal genera within each community. Genera with relative abundances below 1% were grouped into ‘others’. (**A**) Pedicel samples stored at 0 °C; (**B**) berry samples stored at 0 °C; (**C**) pedicel samples stored at 10 °C; (**D**) berry samples stored at 10 °C.

Genus level analyses (Figure 5) focused on those with a relative abundance exceeding 1%. The most abundant classified genus across all samples was *Zygosporium*, with an abundance range of 4.46% to 65.28%. *Cladosporium* was the second most dominant classified genus, with an abundance of 1.20% to 35.74%. Other significant genera included *Aspergillus* (0.01% to 24.33%), *Acremonium* (0.25% to 23.04%), *Podosordaria* (1.04% to 15.68%), *Zasmidium* (0.77% to 13.76%), *Penicillium* (0.02% to 13.83%), and *Alternaria* (0% to 4.40%).

### 3.5. Core Fungal Community of Grape Bunches at Different Storage Temperatures

**Figure 6:**
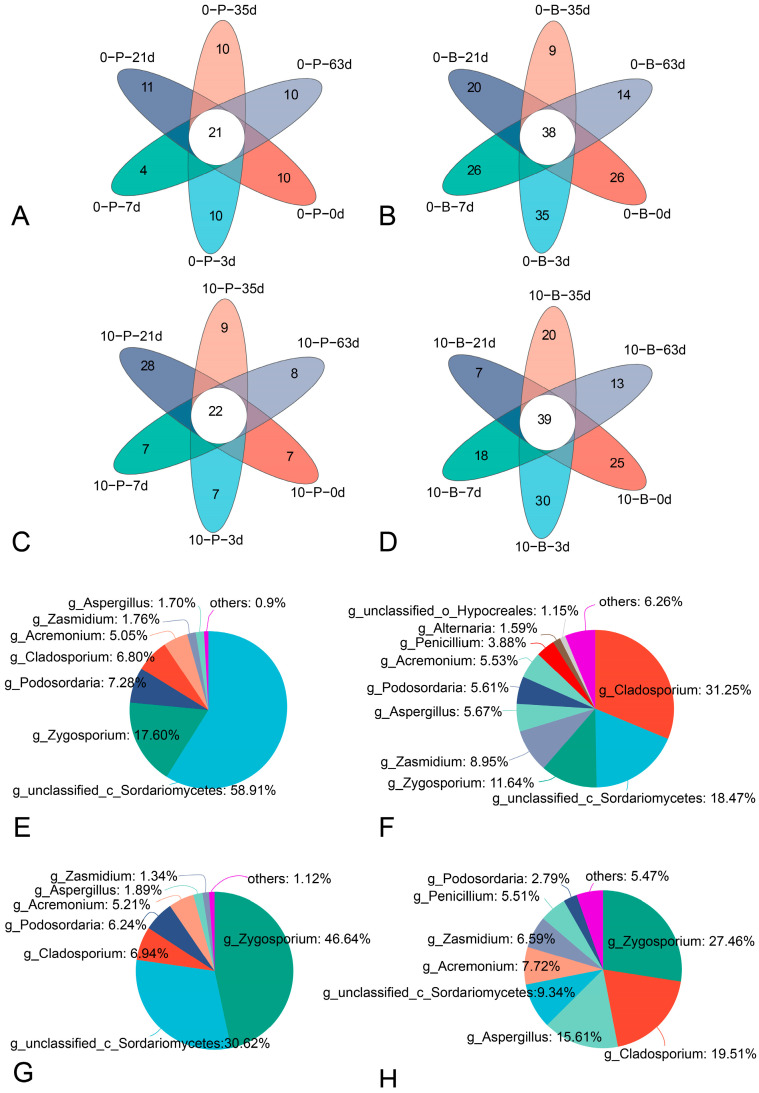
Six-way Venn diagrams of fungal genera and core fungal community composition in grape berry and pedicel samples stored at 0 °C and 10 °C without fungicide treatment. Venn diagrams of the pedicel samples (**A**) and berry samples (**B**) stored at 0 °C and pedicel samples (**C**) and berry samples (**D**) stored at 10 °C. Core fungal community composition in pedicel samples (**E**) and berry samples (**F**) stored at 0 °C and pedicel samples (**G**) and berry samples (**H**) stored at 10 °C.

Four six-way Venn diagrams illustrate the core fungal communities within distinct sample groups (Figure 6). The genera shared by all samples were as follows: 21 in Group 0-P, 38 in 0-B, 22 in 10-P, and 39 in 10-B. Although there is a high degree of consistency in the core fungal genera across these groups, the relative abundance of each genus within the core microbiome varies between groups. The dominant classified genera in these core fungal communities included *Cladosporium*, *Zygosporium*, *Acremonium*, *Podosordaria*, *Penicillium*, *Zasmidium*, and *Alternaria*.

### 3.6. Interactions Among Ecological Communities at Varying Storage Temperatures

**Figure 7:**
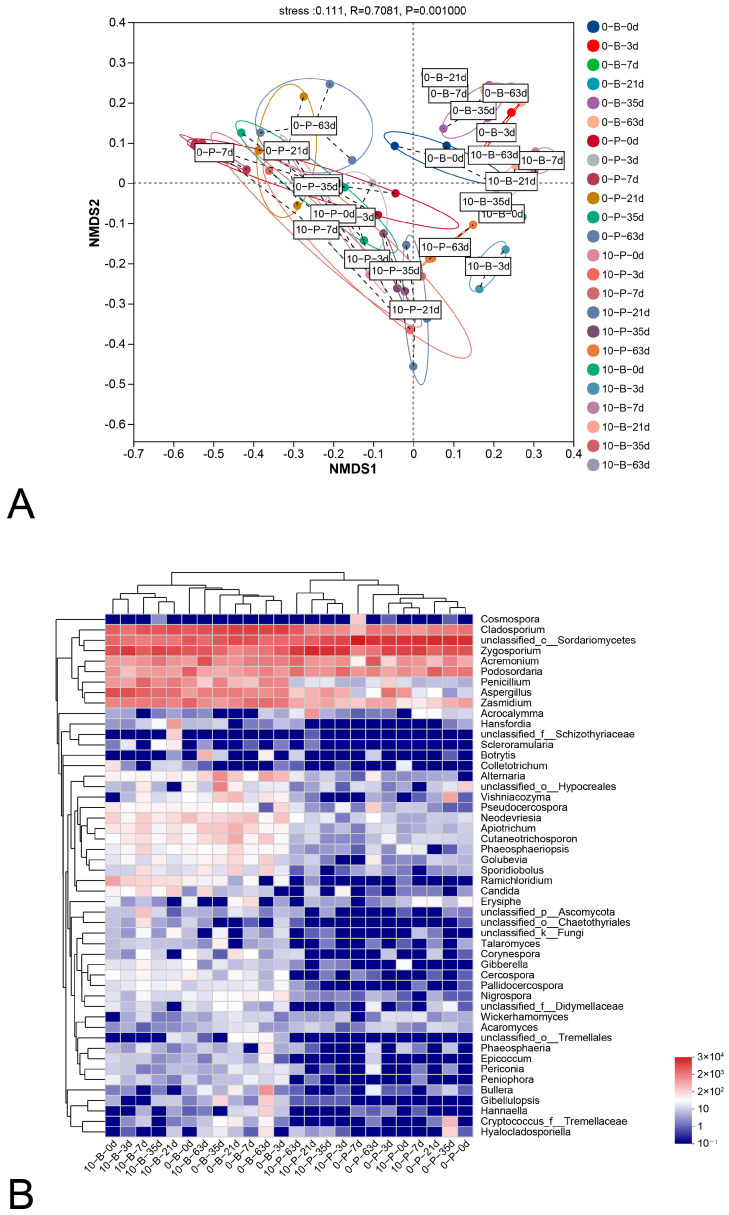
Non-metric multidimensional scaling analysis (**A**) of fungal communities based on genus-level distance at 3%, and a heat map (**B**) of fungal communities based on Bray–Curtis distance indices. Clustering based on Bray–Curtis distance indices was calculated at the genus level with at a 3% distance threshold (located at the top of the heat map). Color intensity on the right-hand panels indicates the relative taxa abundance in percentage, with only the top 50 genera by average abundance shown. For interpretation of color references in this legend, refer to the Web version of this article. 0-B, 0-P, 10-B, and 10-P represent the berry samples stored at 0 °C, the pedicel samples stored at 0 °C, the berry samples stored at 10 °C, and the pedicel samples stored at 10 °C, respectively.

The relationships within ecological communities across various sampling times and storage temperatures were visualized using NMDS (Figure 7A) and a genus level heatmap with hierarchical clustering based on Bray–Curtis distance was generated (Figure 7B). NDMS analysis distinguished samples in two main groups: berry and pedicel. Each of these groups was further separated into subgroups (0-B and 10-B, 0-P and 10-P), indicating distinct fungal compositions between berry and pedicel samples and suggesting that storage temperatures also influenced fungal profiles.

**Figure 8:**
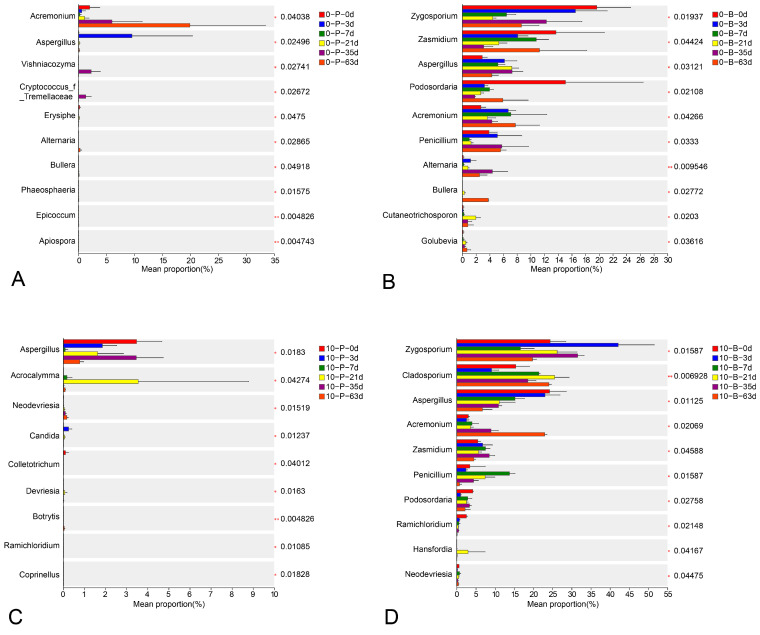
Kruskal–Wallis H test analysis of relative abundances of predominant fungal genera (mean proportion ≥ 1%, showing top 10). Bars represent means ± standard deviation (*n* = 3). (**A**) Pedicel samples stored at 0 °C; (**B**) berry samples stored at 0 °C; (**C**) pedicel samples stored at 10 °C; (**D**) berry samples stored at 10 °C.* *p* < 0.05, ** *p* < 0.01.

The Kruskal–Wallis H test highlighted significant fluctuations in the relative abundances of predominant fungal genera with a mean proportion ≥ 1% over time. For 0-B and 0-B samples, genera such as *Zygosporium*, *Zasmidium*, *Aspergillus*, *Podosordaria*, *Acremonium*, *Penicillium*, and *Cladosporium* showed significant abundance fluctuations (*p <* 0.05) during storage (Figure 8A,B). In the 0-P and 10-P samples (Figure 8C,D), *Aspergillus*, *Acremonium*, and *Acrocalymma* similarly varied in abundance over time.

**Figure 9:**
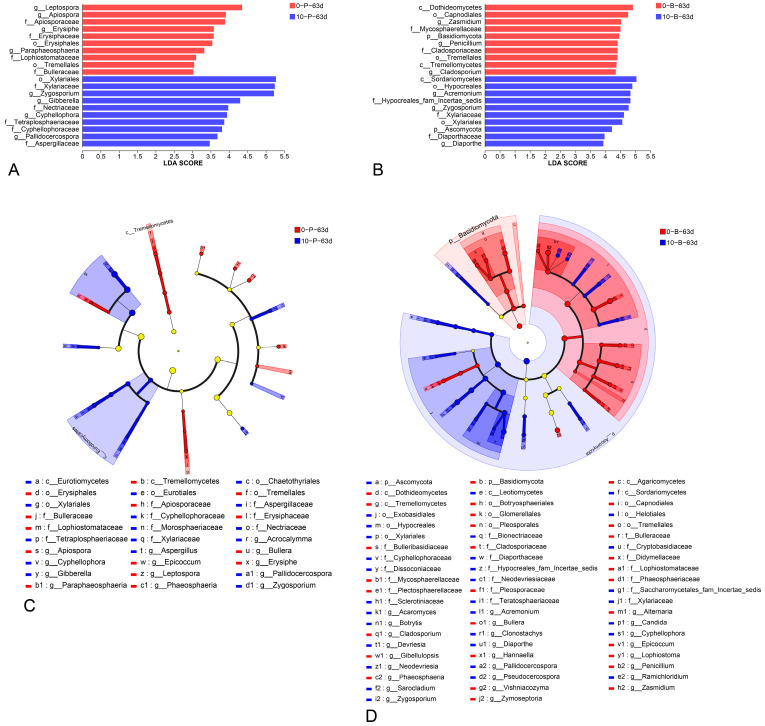
Comparative fungal diversity analysis of berry (0-B-63d and 10-B-63d) and pedicel (0-P-63d and 10-P-63d) samples stored at different temperatures for 63 days, using LEfSe. Histograms of differentially abundant features among (**A**) pedicel sample groups (logarithmic LDA score ≥ 2.0 and *p* ≤ 0.05) and (**B**) berry sample groups (logarithmic LDA score ≥ 2.0 and *p* ≤ 0.05). Taxonomic cladograms highlighting significant differences between (**C**) pedicel sample groups and between (**D**) berry sample groups. Yellow nodes indicate the absence of a statistically significant difference in species composition between the two sample groups. For interpretation of color references in this legend, refer to the Web version of this article.

LEfSe analysis results revealed taxonomic differences between groups at day 63 (0-P-63d vs. 10-P-63d and 0-B-63d vs. 10-B-63d), with statistical significance (*p* < 0.05) and LDA scores ≥ 2.0 (Figure 8). Histograms (Figure 9A,B) and cladograms (Figure 9C,D) show that, for 0-P-63d and 10-P-63d, a total of 41 significantly different taxa, including 2 phylum, 5 classified classes, 8 classified orders, 14 classified families, and 10 classified genera, were identified, with 20 enriched in 0-P-63d and 21 in 10-P-63d. In 0-B-63d vs. 10-B-63d, 65 distinct taxa, including 2 phylum, 8 classes, 13 classified orders, 21 classified families, and 26 classified genera, were identified, with 32 taxa enriched in 0-B-63d and 33 in 10-B-63d. At the genus level, certain genera were notably present in each group. In 0-B-63d, 12 genera (*Cladosporium*, *Zasmidium*, *Zymoseptoria*, *Epicoccum*, *Lophiostoma*, *Phaeosphaeria*, *Alternaria*, *Penicillium*, *Gibellulopsis*, *Bullera*, *Hannaella and Vishniacozyma)* were highly abundant, while 14 genera (*Ramichloridium*, *Pallidocercospora*, *Pseudocercospora*, *Neodevriesia*, *Devriesia*, *Cyphellophora*, *Botrytis*, *Candida*, *Diaporthe*, *Clonostachys*, *Acremonium*, *Sarocladium*, *Zygosporium and Acaromyces)* were abundant in 10-B-63d. In 0-P-63d, seven genera *(Epicoccum*, *Paraphaeosphaeria*, *Leptospora*, *Phaeosphaeria*, *Erysiphe*, *Apiospora*, *and Bullera)* were prevalent, whereas six genera (*Pallidocercospora*, *Acrocalymma*, *Cyphellophora*, *Aspergillus*, *Gibberella*, *and Zygosporium*) were highly detected in 10-P-63d.

## 4. Discussion

The grape variety ‘Shine Muscat’ has become increasingly popular, due to its high nutritional value, distinctive musky flavor, exceptional taste, productivity, and disease resistance. The aroma, particularly the muscat flavor, is a key factor in its market value. Research by Matsumoto and Ikoma [39] demonstrated that post-harvest storage temperature significantly influences this muscat flavor, with lower temperatures accelerating flavor loss and reducing linalool content, while storage at 10 °C helps delay and reduce these effects. Further investigation by Jia et al. [40] unveiled that a reduction in linalool content directly diminishes the muscat flavor in the berries and that the VvHDA15 gene might impact the acetylation of pivotal regulators associated with post-harvest berry quality. Thus, maintaining a temperature of 10 °C for short-term storage exhibits promising potential for preserving the flavor of ‘Shine Muscat’.

Despite limited studies on the microbial community during the cold storage of grape bunches, it is evident that storage temperature, environmental humidity, gas composition, packaging materials, and physical and chemical preservation techniques significantly influence the mycobiota of grape bunches during storage [41]. Elevated temperature and humidity notably increase the incidence of post-harvest diseases caused by decay-inducing fungi. Storing ‘Shine Muscat’ grapes at 10 °C fosters more pronounced growth of spoilage microorganisms compared to storage at 0 °C. During storage at different temperatures, fungal biomarker abundance fluctuates in various ways. Previous research has indicated that table grapes are vulnerable to several key post-harvest diseases at low temperatures (0–5 °C), including blue mold caused by *Penicillium* spp., gray mold caused by *Botrytis cinerea*, and *Alternaria* spot caused by *Alternaria* species. At room temperatures (25–30 °C), grapes are more prone to *Aspergillus* rot caused by *Aspergillus* spp., soft rot caused by *Rhizopus* spp. and black spot caused by *Cladosporium* spp. Studies specific to eastern China found that common post-harvest diseases in ‘Shine Muscat’ grapes include *Alternaria* spot (29.2%), grey mold (20.4%), and *Aspergillus* rot (16.7%) [42]. Abdelfattah et al. found that intensive cultivation practices and frequent fungicide use are associated with a high prevalence of post-harvest pathogens, particularly *Botrytis* and *Cladosporium* species [43]. Carmichael et al. identified *Cladosporium* and *Alternaria* as the pathogenic fungal genera in grape bunches from northeastern South Africa [44]. In this study, at 0 °C, there was no significant change in the abundance of *Cladosporium*, whereas the abundance of *Aspergillus*, *Penicillium*, and *Alternaria* significantly increased from the start of storage to 21 days. This suggests that, during short-term storage, the risk of *Aspergillus* rot, blue mold, and *Alternaria* spot on pedicels is relatively high. At 10 °C, the abundance of *Cladosporium* exhibited a fluctuating yet overall increasing trend throughout the storage period, emerging as the predominant spoilage fungus. *Aspergillus* initially showed a relatively high abundance but this significantly declined over time, indicating that 10 °C storage effectively suppressed the development of *Aspergillus* rot. *Penicillium* abundance peaked at 7 days of storage and subsequently decreased, suggesting its significant impact during the early stages of storage. The main spoilage fungi in this study were similar to those reported previously, but the variation patterns of each genus under different storage temperatures and times differed somewhat from existing reports. Interestingly, *Botrytis* was found at low levels, suggesting a reduced likelihood of gray mold under these storage conditions, contrasting with prior studies where gray mold was the predominant post-harvest disease for table grapes [45,46]. While the post-harvest impacts of *Penicillium* sp., *Aspergillus* sp., and *Botrytis* sp. have been well-documented, diseases caused by *Alternaria* sp. and *Cladosporium* sp. have received less attention [47]. Consequently, preservation techniques specifically targeting black rot will be essential to maintain quality during ‘Shine Muscat’ grape storage at 10 °C.

In addition to typical spoilage fungi, such as *Aspergillus*, *Penicillium*, *Cladosporium*, and *Alternaria*, some genera are known to contain microorganisms that cause spoilage and are more prevalent at 10 °C. For example, *Devriesia* is implicated in sooty blotch and flyspeck on rubber trees and apples [48], while *Diaporthe* species are known to cause serious plant diseases, including cankers and melanosis in citrus [49,50]. These findings suggest that berries stored at 10 °C are at an elevated risk of spoilage from pathogenic microorganisms. However, there is no evidence in the literature indicating that *Zygosporium*, the most dominant genus, causes post-harvest diseases in grapes. The impact of *Zygosporium* on grape bunch quality remains to be elucidated. These fungi primarily originate from soil, the atmosphere, decomposed plant materials, and water sources.

In this study, the α-diversity of fungi showed no significant difference between the 0-B, 0-P, and 10-P groups, while it varied significantly in the 0-B group. These results indicate that a 0 °C low-temperature environment effectively maintained the α-diversity of fungi in both ‘Shine Muscat’ grape berries and pedicels. In contrast, at 10 °C, storage had minimal effects on the fungal diversity of the pedicels but caused significant fluctuations in the fungal diversity of the berries, suggesting that changes in fungal diversity were more sensitive in berries than in pedicels. Existing studies have demonstrated that elevated storage temperatures significantly reduce fungal diversity on the surfaces of fruits such as blueberries and cherries, whereas lower temperatures help maintain community stability [48], which aligns with our findings. To our knowledge, no previous studies have examined changes in fungal diversity on pedicels during storage. We hypothesize that lower diversity and reduced thermal sensitivity of the fungal community on pedicels may be due to the lignified structure of the pedicels and the oligotrophic microenvironment.

In China, the small-scale, labor-intensive nature of ‘Shine Muscat’ grape cultivation often limits growers’ access to specialized preservation equipment. Consequently, many rely on basic refrigeration without fungicides for temporary fruit preservation, which helps alleviate the pressure of immediate harvest and sales. In this study, bunches stored at 0 °C exhibited a decay rate of 1.67% ± 0.89% at 35 days, while those at 10 °C showed a decay rate of 7.33% ± 1.65%. These findings suggest that short-term storage (up to five weeks) at 10 °C is feasible without the use of fungicides, despite the increased spoilage risk.

Previous studies have predominantly focused on fungal communities on grape berries, examining both epiphytic (surface) and endophytic (internal) fungi [51,52,53,54], with limited studies exploring the fungal community on grape pedicels. This study addressed this gap by comparing the microbial community compositions between grape berries and pedicels under varying storage temperatures. Venn diagrams and genus level community bar plots demonstrated greater fruit microbial diversity on the berries compared to the pedicels at both 0 °C and 10 °C. The Kruskal–Wallis H test further indicated a greater number of significantly fluctuating genera in the berry microbiome than in the pedicel microbiome during storage. Key rot pathogens, including *Aspergillus*, *Penicillium*, and *Cladosporium,* were notably affected. These findings suggest that grape pedicels are more resistant to decay during storage compared to berries.

Mycobiota play a critical role in determining the post-harvest health of fruits [44]. DNA metabarcoding, a robust tool for investigating mycobiota, has been extensively utilized in studies focused on post-harvest mycobiota in fruits. For instance, Lane et al. demonstrated that variation in mycobiota during storage was associated with soluble solids concentration and internal browning (IAD), further elucidating the relationship between microbial dynamics and apple quality. Additionally, low-temperature storage was found to reduce the capacity of the indigenous bacterial community to inhibit fungal pathogen colonization [55]. Huang et al. found that changes in the microbial community on the surface of grapes during room-temperature storage conditions were closely associated with disease development, with bacterial community structure alterations being more pronounced compared to those in the fungal community. Fungal β-diversity exhibited a gradual decline during grape storage. On day 4, microorganisms involved in lipid metabolism played a critical role in the post-harvest decay process of grapes [56]. Gao et al. demonstrated that microbial community dynamics during storage were closely related to differences in post-harvest quality and decay in organically and conventionally cultivated pears. After 30 days of storage at room temperature, *Fusarium* and *Starmerella* emerged as the predominant epiphytic fungi on organic pears, whereas *Meyerozyma* predominated on conventionally cultivated pears [57]. In conclusion, with advancement in technologies such as single molecule real-time (SMRT) sequencing [58] and high-throughput absolute abundance quantification [59], the DNA metabarcoding approach is expected to play an even more significant role in characterizing post-harvest fungal communities in agricultural products.

## 5. Conclusions

This study provides a pioneering analysis of fungal community dynamics in ‘Shine Muscat’ grape bunches from a subtropical region during cold storage at varying temperatures, utilizing the DNA metabarcoding approach. The results indicate that fungal diversity is significantly higher on berries than on pedicels. Storage at 0 °C maintains the relative stability of fungal community structures on both berries and pedicels, whereas storage at 10 °C leads to significant changes in the fungal community, particularly on the berries. The fungal community on berries exhibits greater sensitivity to temperature fluctuations compared to that on pedicels. *Aspergillus*, *Penicillium*, and *Alternaria* are the primary spoilage fungi during short-term storage at 0 °C, while *Cladosporium* becomes the dominant spoilage fungus at 10 °C. Although fungicide-free storage at 10 °C increases spoilage risk, it remains a feasible strategy for short-term preservation (up to 5 weeks). To enhance preservation efficacy at this temperature, integrating various physical and chemical preservation methods warrants further investigation. This study lays a foundation for exploring fungal communities on berries and pedicels and their variations under different cold storage conditions. However, deeper insights into the effects of cultivation practices on fungal communities during post-harvest storage and the correlation between fungal communities and grape quality remain areas for future research.

## Figures and Tables

**Table 1:** Fungal composition of samples at the phylum level.

ID	Ascomycota (%)	Basidiomycota (%)	Others (%)	ID	Ascomycota (%)	Basidiomycota (%)	Others (%)
0-B-0d	98.89 ± 0.40	1.08 ± 0.39	0.04 ± 0.04	0-P-0d	99.72 ± 0.15	0.28 ± 0.15	0.00 ± 0.00
0-B-3d	98.70 ± 0.28	1.24 ± 0.28	0.06 ± 0.01	0-P-3d	99.70 ± 0.23	0.30 ± 0.23	0.00 ± 0.00
0-B-7d	98.03 ± 0.49	1.96 ± 0.49	0.02± 0.01	0-P-7d	99.95 ±0.02	0.05 ±0.02	0.00 ± 0.00
0-B-21d	91.97 ± 0.13	7.83 ± 1.22	0.20 ± 0.16	0-P-21d	99.69 ± 0.12	0.31 ± 0.12	0.00 ± 0.00
0-B-35d	95.61 ± 1.02	4.22 ± 1.14	0.16 ± 0.13	0-P-35d	96.08 ± 1.80	3.92 ± 1.80	0.00 ± 0.00
0-B-63d	91.88 ± 1.13	8.12 ± 1.13	0.00 ± 0.00	0-P-63d	99.32 ± 0.37	0.68 ± 0.37	0.00 ± 0.00
10-B-0d	98.69 ± 0.12	1.31 ± 0.12	0.00 ± 0.00	10-P-0d	99.93 ± 0.03	0.07 ± 0.03	0.00 ± 0.00
10-B-3d	98.75 ± 0.73	1.24 ± 0.71	0.01 ± 0.02	10-P-3d	99.94 ± 0.02	0.06 ± 0.02	0.00 ± 0.00
10-B-7d	96.95 ± 0.79	2.87 ± 0.55	0.18 ± 0.24	10-P-7d	99.74 ± 0.21	0.23 ± 0.18	0.03 ±0.04
10-B-21d	98.05 ± 0.70	1.69 ± 0.59	0.26 ± 0.11	10-P-21d	99.70 ± 0.20	0.29 ± 0.20	0.00 ± 0.00
10-B-35d	98.90 ± 0.10	1.08 ± 0.09	0.03 ± 0.02	10-P-35d	99.92 ± 0.04	0.08 ± 0.04	0.00 ± 0.00
10-B-63d	97.54 ± 1.17	2.44 ± 1.19	0.02 ± 0.01	10-P-63d	99.80 ± 0.06	0.20 ± 0.06	0.00 ± 0.00

## Data Availability

The original contributions presented in this study are included in the article/Supplementary Materials. Further inquiries can be directed to the corresponding authors.

## Supplementary Materials

The following supporting information can be downloaded at: https://www.mdpi.com/article/10.3390/foods14071169/s1, Table S1. Sequence information samples of grape berries and pedicels samples during storage. Table S2. Richness and diversity indices by samples (OUT cutoff of 0.03) of grape berries and pedicels during storage. Figure S1: Rarefaction curves of OTUs (Operational Taxonomic Units) clustered at 97% phylotype similarity level in different samples.
