# Supplementary material for: Compositional Shifts in the Mycobiota of ‘Shine Muscat’ Grape (Vitis labruscana Baily × V. vinifera L.) Bunches During Cold Storage at Different Temperatures
Source: Foods. 2025 Mar 27;14(7):1169. doi: 10.3390/foods14071169 (PMC11988345; doi:10.3390/foods14071169)
Supplement: Supplementary file 1 [file foods-14-01169-s001.zip › foods-3489557-supplementary.pdf]

Table S1. Sequence information samples of grape berries and pedicels samples during storage

ID	Number of filtered reads	Number of reads classified as belonging to the fungi kingdom	The minimum length of the ITS sequences (bp)	The maximum length of the ITS sequences (bp)
0-B-0d-1	49268	48730	167	516
0-B-0d-2	60914	60038	185	508
0-B-0d-3	49150	48504	155	527
0-B-3d-1	55932	55134	148	534
0-B-3d-2	54484	53682	187	534
0-B-3d-3	53206	52631	148	520
0-B-7d-1	46452	46148	182	519
0-B-7d-2	46262	45682	148	432
0-B-7d-3	55069	54451	167	528
0-B-21d-1	50753	50625	186	527
0-B-21d-2	52108	52019	186	522
0-B-21d-3	45986	45945	178	512
0-B-35d-1	43198	43078	148	509
0-B-35d-2	49932	49743	185	518
0-B-35d-3	49664	49518	186	521
0-B-63d-1	57497	57214	182	533
0-B-63d-2	59636	59203	182	526
0-B-63d-3	47649	47447	174	519
10-B-0d-1	51924	51784	148	520
10-B-0d-2	48543	47906	188	526
10-B-0d-3	46770	46368	188	485
10-B-3d-1	56946	56421	158	527
10-B-3d-2	50932	50161	160	532
10-B-3d-3	55140	54627	187	515

10-B-7d-1	54613	54510	179	477
10-B-7d-2	43562	43463	142	526
10-B-7d-3	57500	57336	165	526
10-B-21d-1	66124	65547	175	534
10-B-21d-2	62341	61961	170	509
10-B-21d-3	55898	55789	186	516
10-B-35d-1	54141	53946	144	520
10-B-35d-2	53318	52565	193	519
10-B-35d-3	51090	50732	192	527
10-B-63d-1	52525	51980	150	503
10-B-63d-2	51430	51010	148	371
10-B-63d-3	52885	52276	167	494
0-P-0d-1	55665	55617	188	397
0-P-0d-2	39975	39849	187	519
0-P-0d-3	40663	40455	185	518
0-P-3d-1	44214	44077	180	523
0-P-3d-2	39130	38943	188	515
0-P-3d-3	49124	48928	188	529
0-P-7d-1	54298	54163	183	534
0-P-7d-2	54431	54380	185	497
0-P-7d-3	53890	53836	183	505
0-P-21d-1	44617	44479	188	513
0-P-21d-2	45552	45391	188	515
0-P-21d-3	52179	52013	149	529
0-P-35d-1	41886	41602	182	463
0-P-35d-2	38617	38458	184	512
0-P-35d-3	69617	69380	184	467

0-P-63d-1	40520	40402	188	520
0-P-63d-2	62027	61802	166	526
0-P-63d-3	47211	47049	188	524
10-P-0d-1	51910	51728	176	522
10-P-0d-2	40948	40792	187	519
10-P-0d-3	30371	30265	188	526
10-P-3d-1	44814	44703	144	526
10-P-3d-2	37043	36930	188	528
10-P-3d-3	34756	34596	193	538
10-P-7d-1	60516	60388	185	502
10-P-7d-2	37367	37271	188	486
10-P-7d-3	43340	43220	144	498
10-P-21d-1	33944	33865	188	478
10-P-21d-2	41958	41792	188	525
10-P-21d-3	53324	53069	143	501
10-P-35d-1	55171	55013	188	509
10-P-35d-2	36558	36488	183	355
10-P-35d-3	42096	41973	188	524
10-P-63d-1	43238	42987	183	526
10-P-63d-2	42089	41847	188	529
10-P-63d-3	40141	39834	188	538

Table S2. Richness and diversity indices by samples (OUT cutoff of 0.03) of grape berries and pedicels during storage

ID	Reads	OTUs	Diversity and richness index				
			Shannon	ACE	Chao	PD	Coverage
0-B-0d	45434±6820	134.00±16.00	2.3797 ±0.1699	142.2152±17.4185	142.5960±16.6751	33.7565±3.8029	0.9995±0.0000
0-B-3d	44156±3351	142±4.67	2.4918 ±0.1437	149.6788±4.6272	155.2556±1.3926	36.2409±1.8744	0.9995±0.0001
0-B-7d	54206±211	121.0±2.0	2.4033 ±0.1070	124.1275±1.1846	124.3810±1.2540	33.9982±2.2708	0.9997±0.0001
0-B-21d	47449±3153	76.67±12.22	2.5946 ±0.1092	83.7761±13.4963	85.4000±17.7333	22.1808±3.9824	0.9999±0.0001
0-B-35d	50040±13051	77.33±8.89	2.7216±0.1260	78.2208±9.4805	79.3810±10.2540	22.6326±1.4905	0.9999±0.0000
0-B-63d	49919±8072	84.00±4.00	2.6319±0.0486	85.2289±3.6457	84.7333±3.9889	24.3523±0.7539	0.9999±0.0000
10-B-0d	49079±1897	109.67±21.56	2.5593±0.0600	113.5293±22.3661	117.3222±21.6519	30.3277±5.3220	0.9997±0.0001
10-B-3d	54339±2272	119.00±47.56	2.1199±0.1722	123.9739±26.5386	122.0515±26.2126	30.2205±6.5068	0.9996±0.0001
10-B-7d	51891±5553	91.67±16.22	2.9551±0.1671	92.9117±16.2584	93.3667±15.4889	25.5445±5.4666	0.9999±0.0000
10-B-21d	61454±3704	79.67±9.78	2.5525±0.1028	81.7121±10.7467	81.6667±11.1111	23.2628±5.1237	0.9999±0.0001
10-B-35d	52850±1173	98.00±22.00	2.4200±0.0573	101.7890±25.6826	101.7556±25.5407	25.9673±4.1326	0.9997±0.0002
10-B-63d	52280±567	114.00±13.33	2.3933±0.0301	122.2000±17.3672	125.4143±16.7238	28.1814±2.8232	0.9995±0.0002
0-P-0d	45434±6820	48.0±16.0	1.2170±0.6246	62.0631±23.4680	58.9722±22.8148	13.4113±4.6881	0.9996±0.0002
0-P-3d	44156±3351	48.67±7.56	1.4111±0.1424	58.1663±10.5115	56.8167±10.1222	13.8008±3.1181	0.9996±0.0001
0-P-7d	54206±211	33.67±0.44	0.5132±0.1664	43.4223±1.8002	41.5417±2.3056	9.2278±0.9291	0.9997 ±0.0000
0-P-21d	47449±3153	54.67±5.56	1.0989±0.1791	65.6700±7.5488	65.3095±8.4603	16.4984±2.3723	0.9996±0.0001
0-P-35d	50040±13051	46.0±9.33	1.4469±0.2535	54.4640±8.0449	52.0833±11.7222	15.1760±3.700	0.9997±0.0001
0-P-63d	49919±8072	59.67±4.44	1.4704±0.1680	71.9765±2.4515	69.1667±2.5556	16.5697±0.9786	0.9996±0.0000
10-P-0d	41076±7222	50.0±2.0	1.3212±0.0657	70.1643±6.1950	69.7000±8.2000	14.2078±0.9933	0.9995±0.0000
10-P-3d	38871±3962	43.33±3.78	1.2524±0.1715	76.2851±15.2458	56.5635±9.5291	13.5343±1.5652	0.9995±0.0001
10-P-7d	47074±8961	42.0±7.33	0.9892±0.3052	48.5076±9.7576	47.0917±8.5222	13.8150±1.9620	0.9997±0.0001
10-P-21d	43075±6832	67.33±19.78	1.2445±0.3979	84.1198±25.9165	81.8487±22.8459	20.2317±4.3589	0.9994±0.0002
10-P-35d	44608±7042	49.67±2.44	1.3699±0.0988	69.1667±8.1487	66.5667±1.7556	15.6984±0.3674	0.9995±0.0000
10-P-63d	41823±1121	66.0±3.33	1.7001±0.0613	76.5215±5.3115	73.2473±5.2315	19.1574±1.8596	0.9996±0.0001

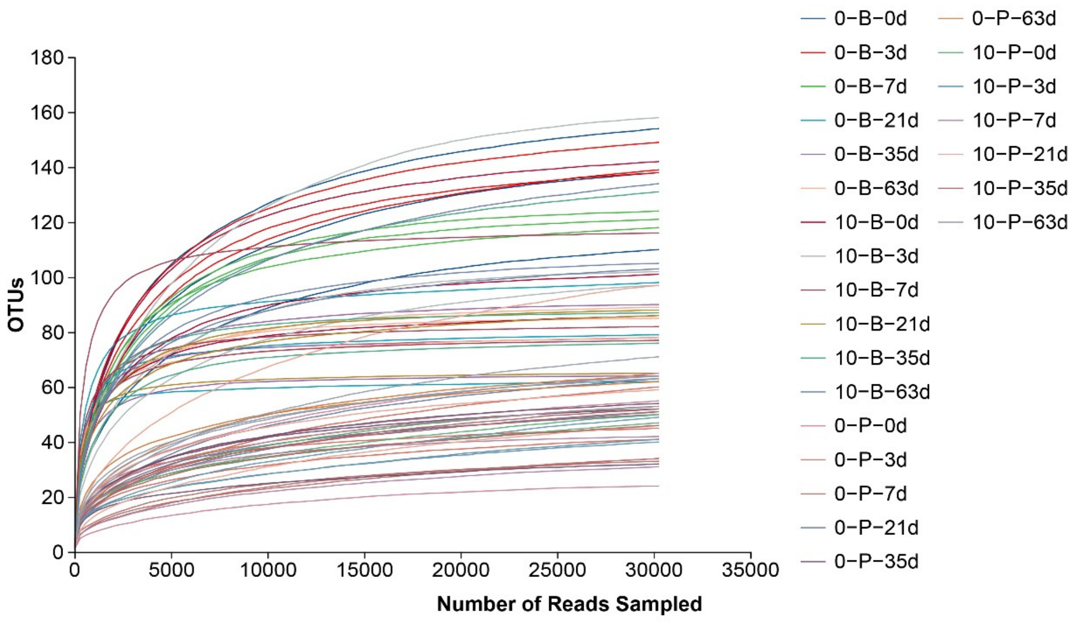

Figure S1. Rarefaction curves of OTUs (Operational Taxonomic Units) clustered at 97% phylotype similarity level in different samples.
